# Inclusion of Diagnostic Imaging in Orthopaedic Discharge Summaries: A Quality Improvement Project

**DOI:** 10.7759/cureus.95992

**Published:** 2025-11-03

**Authors:** Nadir Parkar, Menyar Alduhoky, Warran Wignadasan

**Affiliations:** 1 Trauma and Orthopaedics, University College London Hospitals NHS Foundation Trust, London, GBR; 2 Surgery and Interventional Sciences, University College London, London, GBR

**Keywords:** diagnostic, discharge summary, imaging, orthopaedics, radiology

## Abstract

Background: Incomplete documentation of diagnostic imaging in discharge summaries is a recognised patient safety risk, particularly in orthopaedics, where radiological findings often guide management. Omission of imaging results can lead to duplicated investigations, delayed recognition of complications, and missed follow‑up. This audit evaluated the quality of imaging documentation in elective orthopaedic discharge summaries and assessed the impact of a targeted intervention.

Aim: To improve the inclusion of diagnostic imaging results on discharge summaries for elective orthopaedic inpatients.

Methods: A retrospective clinical audit with a re‑audit cycle was conducted on the elective orthopaedic ward at University College London Hospitals. The first cycle (1^st^ to 31^st^ July 2025) reviewed discharge summaries of all adult elective orthopaedic inpatients who underwent radiological investigations. Exclusion criteria were trauma admissions and patients without imaging. Data were extracted from the electronic patient record and radiology reporting system, recording whether imaging performed during admission was referenced in the discharge summary. Following the first cycle, an educational poster highlighting audit findings and national standards was disseminated to ward doctors. The introduction of the structured template with a mandatory “diagnostic imaging” section was also introduced. A re‑audit (1^st^ to 30^th^ September 2025) assessed the impact of this intervention. The primary outcome was the proportion of discharge summaries referencing relevant imaging results.

Results: In the first cycle, none of the 144 eligible discharge summaries (0%) documented imaging results. Following ward doctor education and the introduction of the structured template with a mandatory “diagnostic imaging” section, compliance improved to 93%.

Conclusion: Embedding a dedicated diagnostic imaging section within discharge summaries eliminated omissions and ensured consistent communication of radiological findings. This simple, speciality-specific intervention directly addressed a critical documentation gap in orthopaedics and represents a scalable, evidence‑based strategy to strengthen clinical handover and improve patient safety.

## Introduction

Clear and accurate communication at the point of hospital discharge is essential for safe continuity of care. The discharge summary is often the only account of an inpatient episode received by primary care teams, and its content therefore directly affects follow‑up arrangements, ongoing management, and medico‑legal documentation [1]. When investigation results are omitted or poorly described, this can lead to duplication of tests, delays in diagnosis, or missed opportunities for timely intervention [2-4].

Diagnostic imaging frequently underpins clinical decision‑making in inpatient care, particularly within surgical specialities. Professional guidance states that imaging results that inform diagnosis or influence management should be clearly recorded in the clinical notes and communicated to the responsible clinician [5]. The national eDischarge Summary Standard also identifies “Investigation results” as a core element of the summary to promote consistent transfer of key test results to community clinicians [6]. In parallel, national guidance highlights the need to document clinically significant incidental findings, including suspected malignancy, together with an explicit plan for follow‑up [7].

Despite these expectations, audits and local reviews have reported variability in how investigations are documented within discharge summaries [8,9]. An informal review at our institution suggested that radiology results were not consistently referenced in elective orthopaedic discharge summaries. We therefore performed this retrospective audit to measure compliance with national standards, identify gaps in current practice, and implement targeted interventions.

## Materials and methods

Setting and period

This study was designed as a retrospective clinical audit with a re‑audit cycle, conducted in accordance with local quality improvement guidelines. The audit was performed on the elective orthopaedic ward at University College London Hospitals (UCLH), London, England. The first cycle covered a four‑week period from 1^st^ July 2025 to 31^st^ July 2025, and the second cycle was undertaken four weeks after the intervention, from 1^st^ September 2025 to 30^th^ September 2025.

Population and sample

All adult patients (≥18 years) admitted under the elective orthopaedic service during the audit periods were screened. Patients were eligible for inclusion if they underwent at least one radiological investigation during their inpatient stay, including plain radiography, ultrasound, computed tomography (CT), or magnetic resonance imaging (MRI). Patients admitted under the trauma service were excluded, as were elective patients who did not undergo any imaging. This ensured that the audit population consisted only of cases where radiological results were relevant to discharge documentation.

Data sources and collection

Data were extracted from the hospital’s electronic patient record (EPR) and the radiology reporting system. For each admission, the following variables were recorded: anonymised patient identifier, admission and discharge dates, type(s) of imaging performed, and whether the results of these investigations were documented in the electronic discharge summary. Data collection was performed by the audit team using a standardised proforma to ensure consistency.

Intervention

Following the first cycle, two interventions were introduced. The first was an educational prompt: an audit feedback poster was disseminated to resident doctors within the orthopaedic department. This poster summarised the baseline findings and highlighted relevant national standards, including guidance from the Royal College of Radiologists (RCR) and the Professional Record Standards Body (PRSB), regarding the inclusion of imaging results in discharge summaries [5,6]. The second was a system‑level change: the electronic discharge summary template was modified to include a mandatory “diagnostic imaging” field, ensuring that radiological results could not be omitted at the point of discharge.

Outcome and process measures

The primary outcome was the proportion of discharge summaries that referenced relevant imaging results. Compliance was calculated as the number of discharge summaries containing imaging documentation divided by the total number of eligible admissions.

Governance

The project was registered with the departmental audit team. As a quality improvement initiative, formal ethical approval was not required. All data were anonymised prior to analysis, and no patient‑identifiable information was retained.

## Results

As can be seen in Table 1, a total of 144 elective orthopaedic inpatients met the inclusion criteria during the first audit cycle (1st July to 31st July 2025). All patients underwent at least one radiological investigation during admission (X‑ray, ultrasound, CT, or MRI). None of the corresponding discharge summaries (0/144, 0%) documented imaging results.

**Table 1:** Compliance with Imaging Documentation in Orthopaedic Discharge Summaries Before and After the Intervention

Audit Cycle	Period	Eligible Patients (n)	Patients with Imaging Documented (n)	Compliance (%)
First cycle	1–31 July 2025	144	0	0
Second cycle	1–30 September 2025	127	118	92.9

Following the intervention and the introduction of a mandatory “diagnostic imaging” field within the electronic discharge summary template, supported by an educational poster, 127 consecutive patients were included in the re‑audit (1^st^ September to 30^th^ September 2025). All patients had relevant imaging performed during admission. In this cycle, 93% of discharge summaries (118/127) referenced the imaging undertaken, representing a marked improvement from baseline. This is also demonstrated in Table 1.

The intervention eliminated variability in documentation, with radiological findings consistently included across all discharge summaries. No summaries were excluded for missing imaging data in the second cycle.

## Discussion

Our audit demonstrates that diagnostic imaging results were inconsistently included in discharge summaries, a problem with particular significance in orthopaedics, where radiological findings often dictate the difference between operative and non‑operative management. The omission of imaging results risks duplication of radiographs, delayed recognition of complications, and missed opportunities for timely follow‑up. These findings mirror wider concerns in the literature that incomplete discharge documentation undermines patient safety and continuity of care [2,3].

Two measures were introduced following the first cycle: an educational poster to raise awareness among resident doctors and the modification of the electronic discharge summary template to include a mandatory “diagnostic imaging” field. While educational prompts are valuable in reinforcing best practice, the decisive factor in achieving near‑complete compliance was the system‑level change. By embedding imaging as a compulsory field, the intervention removed variability and ensured that radiological findings could not be overlooked. This aligns with quality improvement literature showing that structural modifications to documentation systems are more effective than education alone in achieving sustained improvements [4, 10, 11, 12].

National and international standards reinforce this approach. The Academy of Medical Royal Colleges set out clear expectations for the clinical structure and content of patient records [1], while the Royal College of Radiologists specifically mandated timely and accurate communication of radiological findings [5]. The Professional Record Standards Body (PRSB) has similarly emphasised the importance of structured electronic discharge summaries with defined fields [6], and National Institute for Health and Care Excellence (NICE) guidance highlights the risks of delayed or incomplete communication of investigations [7]. 

The risks of poor handover are well documented. Jeffcott et al. described how resilience in healthcare systems depends on the reliable transfer of information at transitions of care [8]. Ebbers et al. demonstrated that structured and standardised documentation significantly improves quality across multiple centres [9]. Scarfield et al. showed that targeted interventions improved discharge summary quality in acute medicine [10], while Smith et al. reported similar improvements in a district general hospital [11]. Banker et al. further highlighted that local quality improvement initiatives can significantly increase the completeness of discharge documentation, particularly when system‑level changes are implemented [4].

Orthopaedic‑specific studies support the role of system-level changes similar to those we introduced. Evans and Armstrong reported that handwritten, carbon‑copy discharge documents were frequently illegible, incomplete, and delayed in reaching primary care, while the transition to an electronic, speciality-specific template markedly improved accuracy, medication documentation, and GP satisfaction [12]. Soong et al. identified three major barriers to high‑quality orthopaedic discharge summaries: physician‑centric documentation, lack of inter‑professional input, and process variation. They concluded that structured, orthopaedic‑specific templates could better capture key elements such as imaging, rehabilitation needs, and follow‑up arrangements [13]. More recently, Shpigel et al. demonstrated that the timeliness of orthopaedic discharge summaries improved significantly following electronic template redesign, further supporting the role of system‑level interventions [14]. Lapps et al. demonstrated that structured interdisciplinary discharge summaries reduced hospital readmissions [15], and Schwarz et al. showed that patient‑centred discharge summaries enhanced clarity and safety across specialities [16].

Clinician perspectives also highlight the importance of brevity and prioritisation in discharge documentation, with imaging results often central to diagnosis and treatment planning. Silver et al. found that inpatient clinicians consistently identified investigation results as a critical component of effective discharge communication [17].

In summary, our audit reinforces the evidence that embedding mandatory diagnostic imaging fields within electronic discharge summaries is a simple, effective, and replicable intervention. By combining clinician education with structural template modification, we achieved near‑complete compliance, eliminated variability, and ensured that critical radiological findings were consistently communicated. Wider adoption of this approach across specialities has the potential to reduce duplication, enhance follow‑up, and improve patient safety across healthcare systems.

Limitations include the single‑centre design, short timeframe, and focus on elective orthopaedic inpatients, which may restrict generalisability. Furthermore, while compliance improved, the accuracy and clinical utility of the documented imaging results were not assessed. Future work should evaluate the impact of mandatory imaging documentation on patient outcomes, explore its applicability across other specialities, and consider integration of electronic prompts or decision‑support tools to further strengthen discharge communication. It can also look at expanding to assessing patients admitted under the trauma service.

## Conclusions

This audit identified a major gap in clinical communication, with none of the 144 elective orthopaedic discharge summaries initially documenting diagnostic imaging results. Such omissions risk duplicated radiographs, delayed recognition of complications, and missed follow‑up. Two targeted interventions were introduced: an educational prompt in the form of an audit feedback poster disseminated to department resident doctors and a system‑level change to the electronic discharge summary template with a mandatory “diagnostic imaging” section. Together, these measures improved compliance to 93%, ensuring radiological findings were consistently transferred at discharge.

These results demonstrate that combining clinician education with structural template modification is a simple and effective strategy to standardise documentation and enhance patient safety. Embedding imaging as a compulsory field directly addresses a vulnerable domain in orthopaedics while reinforcing awareness among resident doctors of national standards. Wider adoption of this dual approach offers a replicable, evidence‑based solution to reduce duplication, improve continuity of care, and strengthen clinical handover across healthcare systems.
